# More than half of ESBL-E are susceptible to fluoroquinolones: admission prevalence data from eight non-ICUs in a German university hospital

**DOI:** 10.1186/2047-2994-4-S1-P124

**Published:** 2015-06-16

**Authors:** F Maechler, N Thoma, P Dem, A Kola, S Hansen, P Gastmeier

**Affiliations:** 1 Institute of Hygiene, Charité, Berlin, Germany

## Introduction

Little information is available on prevalence of extended-spectrum-betalactamase-producing *Enterobacteriaceae* (ESBL-E) with and without additional resistance to fluoroquinolones. In Germany, *Enterobacteriaceae* are classified according to their susceptibility to four classes of antimicrobial substances. Only organisms resistant to acylureidopenicillins, 3^rd^ and 4^th^ generation cephalosporins and fluoroquinolones are labelled as “multidrug–resistant”.

## Objectives

The aim of this prospective analysis was to gain evidence on admission prevalence and incidence of ESBL-E with and without resistance to fluorochinolones in non-ICUs in a German university hospital.

## Methods

This analysis is part of the R-GNOSIS framework. WP 5 investigates the benefits of isolation precautions over standard measures for ESBL-E-carriers in non-ICUs. Rectal swabs are obtained for all patients admitted to the participating wards within 3 days of admission. Patients staying longer than 3 days are screened every 7 days thereafter and before discharge. Chromogenic culture media are used for ESBL-screening, identification and susceptibility testing is performed using Vitek 2 (bioMérieux, Germany).

## Results

Between February 2014 and February 2015, 8317 patients were admitted to 8 medical and surgical wards. An admission sample was obtained for 6047 patients (73%). Among all 8317 patients, 6814 patients had a LOS of more than 3 days, and 4083 patients were screened at least twice (60%).

The majority of ESBL-E-carriers was identified on admission (n=607, 10.1%). However, 197 patients (4.8%) were screened negative on admission and turned ESBL-positive during their stay. Admission prevalence of ESBL-E resistant to fluorochinolones was 4.7% (n=286), and 2.7% (n=111) of patients turned positive during their stay.

## Conclusion

More than 50% of ESBL-E were susceptible to fluorochinolones. As the increase of ESBL-E is a worldwide concern and resources to prevent their spread are limited, focusing on ESBL-E according to their antimicrobial susceptibility pattern may be a pragmatic approach.

## Disclosure of interest

None declared.

